# “You can’t die here”: an exploration of the barriers to dying-in-place for structurally vulnerable populations in an urban centre in British Columbia, Canada

**DOI:** 10.1186/s12904-024-01340-7

**Published:** 2024-01-10

**Authors:** Kelli I. Stajduhar, Melissa Giesbrecht, Ashley Mollison, Kara Whitlock, Piotr Burek, Fraser Black, Jill Gerke, Naheed Dosani, Simon Colgan

**Affiliations:** 1 Institute on Aging and Lifelong Health, University of Victoria, 3800 Finnerty Road, Victoria, BC V8P 5C2 Canada; 2 Canadian Institute for Substance Use Research, University of Victoria, 3800 Finnerty Road, Victoria, BC V8P 5C2 Canada; 3 Faculty of Medicine - Island Medical Program, University of British Columbia, 3800 Finnerty Road, Victoria, BC V8P 5C2 Canada; 4 Palliative and End of Life Care Program, Vancouver Island Health Authority, 1952 Bay Street, Victoria, BC V8R 1J8 Canada; 5 Palliative Care Physician, Department of Family & Community Medicine, St Michael’s Hospital at Unity Health Toronto, 36 Queen St E, Toronto, ON M5B 1W8 Canada; 6 Cumming School of Medicine, University of Calgary, 2500 University Dr. NW, Calgary, AB T2N 1N4 Canada

**Keywords:** Canada, Dying in place, End-of-life care, Ethnographic methods, Health equity, Homelessness, Structural vulnerability, Thematic analysis

## Abstract

**Background:**

One measure of quality in palliative care involves ensuring people approaching the end of life are able to receive care, and ultimately die, in the places they choose. Canadian palliative care policy directives stem from this tenet of autonomy, acknowledging that most people prefer to die at home, where they feel safe and comfortable. Limited research, however, considers the lack of ‘choice’ people positioned as structurally vulnerable (e.g., experiencing extreme poverty, homelessness, substance-use/criminalization, etc.) have in regard to places of care and death, with the option of dying-in-place most often denied.

**Methods:**

Drawing from ethnographic and participatory action research data collected during two studies that took place from 2014 to 2019 in an urban centre in British Columbia, Canada, this analysis explores barriers preventing people who experience social and structural inequity the option to die-in-place. Participants include: (1) people positioned as structurally vulnerable on a palliative trajectory; (2) their informal support persons/family caregivers (e.g., street family); (3) community service providers (e.g., housing workers, medical professionals); and (4) key informants (e.g., managers, medical directors, executive directors). Data includes observational fieldnotes, focus group and interviews transcripts. Interpretive thematic analytic techniques were employed.

**Results:**

Participants on a palliative trajectory lacked access to stable, affordable, or permanent housing, yet expressed their desire to stay ‘in-place’ at the end of life. Analysis reveals three main barriers impeding their ‘choice’ to remain in-place at the end of life: (1) Misaligned perceptions of risk and safety; (2) Challenges managing pain in the context of substance use, stigma, and discrimination; and (3) Gaps between protocols, policies, and procedures for health teams.

**Conclusions:**

Findings demonstrate how the rhetoric of ‘choice’ in regard to preferred place of death is ethically problematic because experienced inequities are produced and constrained by socio-structural forces that reach beyond individuals’ control. Ultimately, our findings contribute suggestions for policy, programs and practice to enhance inclusiveness in palliative care. Re-defining ‘home’ within palliative care, enhancing supports, education, and training for community care workers, integrating palliative approaches to care into the everyday work of non-health care providers, and acknowledging, valuing, and building upon existing relations of care can help to overcome existing barriers to delivering palliative care in various settings and increase the opportunity for all to spend their end of life in the places that they prefer.

**Supplementary Information:**

The online version contains supplementary material available at 10.1186/s12904-024-01340-7.

## Introduction

Place, particularly ‘where’ one dies, has become a proxy measure of quality palliative and end-of-life care [1–5]. However, there is a lack of research that critically explores how place might be experienced differently by those who have largely been excluded from traditional palliative care research and services, for example people experiencing extreme poverty, people unstably housed, and/or people who use illicit substances [4]. Palliative care refers to the prevention and relief of suffering for people who are experiencing life-threatening illness, including their caregivers and/or family members, through the holistic approach of physical, psychosocial, and spiritual care and support [6]. Across much of the global north, including Canada, policy has shifted towards the promotion of ‘choice’ in regard to where people would like to receive palliative care, and ultimately die [3, 7, 8]. The impetus for this policy directive stems in part from the idea that the majority of people would prefer to die at home, where they feel most comfortable and connected to the people and places that are meaningful to them [5, 7, 9]. Although the discourse of ‘choice’ regarding preferred place of death is commonly used, it is underpinned by the assumption that everyone has the same opportunities to make autonomous choices that will allow them to achieve the deaths they want, in the places they want. What fails to be acknowledged, however, is how social and structural forces influence opportunities and choice with respect to palliative care, including place of death [2, 4, 10, 11].

Everyone approaching their end of life will likely experience increased vulnerability due to their need for care [12], however, this vulnerability is compounded further by their deprivation in relation to social and structural determinants of health [13–15]. Structural vulnerability is an analytic concept that captures how populations’ freedoms and opportunities are constrained due to their lower ranking in the social hierarchies [13, 16]. This positioning is the outcome of various structural processes of oppression (e.g., racism, classism, colonialism) that taken together, serve to amplify vulnerability to risk, harm, and negative health outcomes [16–18]. In our research (e.g., [14, 15, 19, 20]), we have identified structural vulnerability among populations experiencing social isolation, racism, trauma, violence, and stigma associated with living in poverty and experiencing various levels of homelessness in conjunction with multiple intersections of mental health issues, including cognitive impairments, behavioural issues, previous or ongoing substance use, experience with the criminal justice system, and/or mobility challenges. We have also witnessed how people discriminated by and excluded from mainstream society meet their own health and social needs by forming communities of care and support, yet these strategies are often not recognized nor well understood, and sometimes actively suppressed [21].

Structurally vulnerable populations will most likely have a greater need for care due to the degree of systemic suffering, deprivation associated with determinants of health, and resulting negative health outcomes [13, 14, 22–24]. However, they face disproportionate barriers in accessing needed care, particularly at the end of life [13, 14, 22–24]. The critical need to prioritize access to food/shelter while navigating complicated health/social care systems [14, 15] for example, creates barriers in accessing care. In addition, the ability to seek out or accept care at the end of life by structurally vulnerable populations tends to be highly dependent upon whether it is considered safe to do so (e.g., ‘who’ is providing such care and ‘where’). For example, research has demonstrated how people who have largely been excluded, or previously mistreated, by traditional health and social care systems avoid seeking medical care at the end of life due to mistrust of medical professionals. Previous experiences of discrimination, racism, and being deemed unworthy by providers to receive life-sustaining treatments will also significantly impact access [23, 25, 26]. Additionally, formal institutional settings, like hospitals, are avoided by structurally vulnerable populations because of the stigmatization, social exclusion, and power imbalances inherent within these spaces [20, 27, 28].

Considering the barriers mentioned above, ensuring structurally vulnerable populations the opportunity to die-in-place, defined here as receiving quality palliative care and supports by people they trust in the places they feel safe (e.g., in their homes/communities), becomes critically important. This involves bringing palliative care to their communities and into their homes, however it is defined or experienced by them (e.g., shelters, transitional housing units, etc.) [26, 29–31]. However, little research has explored how people positioned as structurally vulnerable experience dying-in-place. As such, we draw from longitudinal data collected across two studies that took place from May 2014 to December 2019 in an urban centre in western British Columbia. After reviewing the findings that emerged from each of these studies, it became apparent that, due to multiple complex challenges, people positioned as structurally vulnerable do not have the options or ‘choice’ to receive end-of-life care and die-in-place. By revisiting our complete data set, including ethnographic fieldnote, interview, and focus group data, we examined the barriers and challenges that appeared to prevent people who experience social and structural inequity from dying-in-place. The purpose of this analysis is not only to illuminate how structural factors constrain ‘choice’ in regard to dying-in-place, but to generate findings that can assist in developing more equitable and inclusive policy, programs, and practices that can improve people’s ability to receive quality palliative care, and ultimately, die in the places that they choose to.

## Methods

This longitudinal qualitative analysis draws from data collected during two studies that took place across the span of over five years, from May 2014 to December 2019, in an urban centre in western British Columbia. In the first Equitable Access to Care (EAC) study, we applied a critical ethnographic approach to examine access to health care for structurally vulnerable populations at the end of life (for more details, see [19, 32]). Research employing ethnographic methodologies seeks to qualitatively explore the nature of particular phenomena within the natural environments that they occur [33–35]. Ethnographers working from critical perspectives seek to address processes of inequity and injustice, and therefore aim to examine not only how power, social structures, and ideologies constrain individual experiences, but also to generate practical knowledge that can influence change [36].

Our second study, Integrating a Palliative Approach to Care in the inner-city (iPAC-IC), grew out of our first and employed a community-based participatory action research approach to develop strategies for integrating a palliative approach to care into the daily work of community service workers who are already providing care for these populations (for more details, see [21]). Community-based participatory action is an approach to research that involves researchers and community stakeholders collaborating as partners in all steps of the research process with the goals of educating, improving practice, and/or bringing about social change [37].

The interdisciplinary research teams for both studies consisted of academics, medical professionals, and community service workers. Ethics was obtained from University of Victoria and Vancouver Island Health Authority Joint Research Ethics Sub-Committee, the University of Victoria Human Research Ethics Board, and Health Research Ethics Board of Island Health.

### Participant overview and data collection

#### EAC study

Participants sought for the initial EAC study comprised of four groups: (1) people positioned as structurally vulnerable who were on a palliative trajectory; (2) their informal support persons/family caregivers (e.g., street family); (3) their formal service providers (e.g., housing workers, medical professionals); and (4) key informants (e.g., managers, medical directors, executive directors). Participant recruitment began with inviting local health, housing, and social care service providers to participate in our study via pamphlets, posters, and presentations that were circulated in their places of employment. To capture maximum participant variation among formal service provider participants, diverse characteristics were purposely sought (e.g., disciplinary background, employment setting) [38]. After interested service providers consented to participate, they were asked to facilitate recruitment of participants facing health and social inequities by identifying and sharing study information with clients whom were on a palliative trajectory. Some structurally vulnerable participants had support persons/family caregivers who provided them with various levels of support and they were also invited to participate. Written consent was initially obtained by all participants at the outset of their involvement in the study and then verbally confirmed throughout the data collection process.

Overall, this study included 25 participants experiencing structurally vulnerability, 25 informal support persons, 69 formal service providers, and 20 key informants. Data collection occurred over a time period of 30 months and involved repeated participant observation with structurally vulnerable participants (and support persons if present) and their interactions with formal service providers. In total, 300 h of observation were conducted in homes, shelters, transitional housing units, at health care appointments, in community-based service centres, and on the street. Observations were conducted without restrictions; at all hours of the day and all days of the week. The goal of observations was to garner data pertaining to experiences of structurally vulnerable populations on palliative trajectories, particularly in regard to their access to health care services. Observational data were supplemented with semi-structured in-depth interviews, which clarified and validated observations. Supplementary Material 1 provides the ethnographic observation and interview guides.

#### iPAC-IC study

Participation for the second study (iPAC-IC) involved recruiting community service workers who had been engaged in our first study and were identified as playing critical roles in the provision of care for structurally vulnerable populations at the end of life. These participants (*n* = 18) included housing and shelter workers, outreach, support, and peer workers, and along with a team of academic researchers, became the community-based action team. During initial action team meetings, the study aim was co-defined, which was to develop strategies for integrating palliative approaches to care into everyday inner-city work while identifying barriers to its implementation. From here, a series of ‘action cycles’ (n = 18) occurred, which involved multiple team meetings focussing on identifying priorities, developing action plans, recruiting and engaging with secondary participants identified as key stakeholders, and community interventions (for more details, see [39]). Primary and secondary participants sought permission from their organizations to participate during paid working hours or received an honoraria of $50 if they participated outside of work time. Participants provided evaluative feedback on any learnings, emerging ideas for practice, and remaining questions/concerns at the end of each team meeting, which in turn informed proceeding meeting agendas, community interventions, and ultimately, the direction of this research study. For instance, when action team members identified the need to ‘know who to call’ when their peers/clients showed up unconscious in hospital, a worker participant supported by the research team led a series of community workshops to create an adapted Advance Care Planning tool and guide [40].

In total, primary participants comprising our action team (*n* = 18) represented six unique local housing and health support organizations and the health authority. Secondary participants (*n* = 48) engaged through our action cycles represented various local health, palliative, social care, and housing organizations. Demographic information was collected from all primary and secondary participants. We also conducted focus groups (*n* = 5) with primary and secondary participants and evaluative interviews (*n* = 13) with primary participants at the end of the study. Structured observational field notes (*n* = 34) were collected during every team meeting and community intervention. Supplementary Material 2 provides the various guides used to collect this iPAC-IC data.

### Analytic process

Interview and focus group data from both studies were digitally recorded and transcribed verbatim, and together with the observational fieldnotes, entered into NVivo™ for analysis. Each data set had previously undergone interpretive thematic analysis, which involved researcher immersion into the data in order to identify common emergent themes that resonated with the research question of focus [41]. Thematic coding schemes for each study were co-developed by the teams, which involved reviewing randomly selected data and then meeting to discuss interpretations of the data until consensus was reached on the themes identified. Emerging themes were then compared, contrasted, reorganized, and refined through a process of data coding, which was conducted by two researchers (MG and AM) [42].

In each of these studies, the theme of ‘barriers to care in the community’ emerged as a significant thematic finding and was coded accordingly. As such, we extracted and combined these coded data to thematically analyze them more in-depth. We began this thematic analysis by having the team review 25 pages of randomly selected coded data, with the aim to identify emerging themes within the ‘barriers to care in the community’ data. From here, a refined thematic coding scheme was collectively developed and then applied to the data using NVivo™ by one research team member (MG). Analysis revealed that multiple complex barriers were constraining the ‘choice’ of structurally vulnerable populations to die-in-place. To enhance analytic rigor, numerous team meetings were held throughout the entire coding and analytic process in order to ensure consensus was met. This process of investigator triangulation enhanced the validity and completeness of the analytic findings, and ensured that all data was coded appropriately into their relevant themes [43].

### Findings

All participants on a palliative trajectory lacked access to stable, affordable, or permanent housing. As a result, they were living in single room occupancy hotels, supportive housing facilities, shelters, or living rough (e.g., tent, boat). Although not ‘traditionally’ defined as homes, the majority of structurally vulnerable participants expressed their desire to stay ‘in-place’ at the end of life, surrounded by familiar environments and trusted support people. Thematic analysis revealed three main barriers impeding their ‘choice’ to remain in-place at the end of life: (1) Misaligned perceptions of risk and safety; (2) Challenges managing pain in the context of substance use, stigma, and discrimination; and (3) Gaps between protocols, policies, and procedures for health teams. It is important to emphasize that all three of these thematic findings are significantly interconnected, however, we have attempted to untangle them here in order to present each facet in greater depth. To ensure participant anonymity, pseudonyms have been used.

### Misaligned perceptions of risk and safety

Our first thematic finding interconnects, to various degrees and in differing ways, to almost all barriers identified, and is perhaps better described as an overarching umbrella. This theme captures the vast misalignment that exists between institutional, organizational, and embodied experiences and perceptions of risk and safety. Analysis revealed the various ways, and depth to which, institutional power is embedded and enacted through the definitions and perceptions of ‘risk’ that take precedence. For example, in response to ‘risks’ defined by the institution, ‘worker safety policies and regulations’ have become implemented by local community health care organizations, with many prohibiting community care nurses or aides from entering ‘high risk’ homes or settings where people positioned as structurally vulnerable often live. Participants shared multiple examples of how people experiencing structural vulnerability were unable to access home care services because of policies pertaining to issues of cleanliness/hygiene (e.g., a broken dish on the floor), the presence of cigarette smoke, alcohol, criminalized drugs or drug-use equipment, overcrowding, or because of a previous report of a violent incident. Service providers and structurally vulnerable participants described how these policies were rooted in population and place-based assumptions. These policies were also based on assumptions regarding the personal risk thresholds of individual community nurses who might not actually feel at ‘risk’ in some of these homes. As a result, ‘choice’ for not only participants positioned as structurally vulnerable, but also their community health care providers, becomes considerably restrained. A palliative care physician described how they were unable to provide chemotherapy at a client’s home because of the system’s “*assumptions that people are living in places and spaces that wouldn’t be safe, I just did air quotes ‘safe’”*. While acknowledging that everyone’s safety is important, many participants expressed frustration over these ‘blanket’ institutional safety policies. Participants shared how these blanket policies resulted in dire circumstances for many declining and dying people, who were left with no choice but to move into an institutional setting (e.g., hospital) to receive needed care, amplifying *their risk* for discrimination and social harms, and resulting in additional suffering.

While many community service worker participants reported cases of clients’ homes being deemed unsafe, it was also observed that entire buildings, and even entire neighborhoods, had been identified as “*unsafe environments*”. Community health care nurses and aides, for example, were restricted from entering many single room occupancy hotels, supportive housing facilities, or shelters, even if the client or client’s home was not considered high risk. Community health care offices and providers were found to have designated particular buildings/city blocks as “*no go*” places, despite no formal criteria for how or why these places were designated as such. Many structurally vulnerable participants remarked on these place-based barriers by describing how difficult it was to get community health nurses and support workers into their neighborhood and how local health care offices were “*hesitant”* to provide care there, especially after dark. While implemented to keep community health providers safe, these place-based boundaries serve to reinforce structural stigma, impede access to care, and restrict structurally vulnerable populations from remaining in their homes, where they may feel safest, at the end of life.

Adding to the complexity, structurally vulnerable participants’ perceptions of risk were also found to influence their willingness to accept professional health care providers into their private homes. Due to experiences of systemic stigmatization and violence, discrimination, and social harms, many participants understandably carried a significant mistrust of the medical system. Participants positioned as structurally vulnerable remarked how they experienced discrimination and *“judgements”* by nurses. For example, ‘George’ shared in an interview that there was only one home care nurse that he allowed into his home:

*Well, he [home care nurse] was efficient and didn’t make a judgement on how messy the place was…he didn’t make an issue of that and he didn’t make a judgment on it, he didn’t make a judgement on me. And also, I live like this and I know I’m a mess, periodically I clean up, but I don’t like to be judged on that, you know? And, um, a friend of mine said I’m ‘not going to win a contest in home care’, you know?*

Accepting home care workers into the home, therefore, put dying participants at greater risk of discrimination and further harms. As health deteriorates and participants became increasingly vulnerable, however, their need for care in the home increased. Desperate to remain at home, some participants were found doing whatever they could to make nurses and support workers “*feel safer”.* For example, one dying structurally vulnerable participant, ‘Sammy’, exerted significant energy cleaning his home, despite his decreased mobility and advanced lung disease, so the he could meet the systems’/workers’ standards of cleanliness. Taken together, these misaligned notions and perceptions of risk and safety experienced simultaneously by institutions, organizations, providers, and clients have resulted in a significantly challenging context to deliver palliative care, and ultimately, for people positioned as structurally vulnerable, to die-in-place.

### Challenges managing pain in the context of substance use, stigma, and discrimination

The option and desire to remain at home at the end of life is often dependent on having pain and other symptoms adequately managed. However, complexly intertwined with our previous finding regarding risk and safety, many barriers to having pain needs met were found to be related to ‘risks’ associated with people who have a history of using, or actively use, illicit substances. Findings indicate that the context of substance use prevented people positioned as structural vulnerable from managing their pain adequately at home, denying them the opportunity or ‘choice’ to die-in-place. For example, barriers to pain management in the community often involved health care professionals’ assumptions of, and fear regarding, the risks associated with drug-seeking and diversion. People positioned as structurally vulnerable frequently described that when they sought out care due to pain that they were experiencing, they were met with discrimination, stigmatization, and assumptions that they had ‘ulterior motives’ and were ‘drug seeking’. During a focus group, these participants shared:

Peer outreach worker: “*you see them back the next day and they’re still in pain, and then they say, “I went* [to the hospital], *but they pretty much told me to go home and gave me an Aspirin or whatever.”* Shelter worker: *Or they’ll say, “you’re drunk” or “you’re high,” and boot them out.* Peer outreach worker: *Exactly! And drug seeking or whatever! And it’s not the case. They’re definitely in need, at least for investigation of what the hell is wrong with them…*Shelter worker: *They need medicine.*

After repeatedly being labelled as ‘drug seeking’, it was shared that structurally vulnerable participants were reluctant to continue trying to access needed care, “*finding it easier to not even go in the first place*” (palliative care nurse). Lifetime experiences such as these within the medical system resulted in increased risks of harm for many dying structurally vulnerable participants, who as a result, tended to avoid all formal health care, including home care, until their pain became completely unbearable. As ‘Harvey’ stated in an interview:

*I am the kind of person that waits till the shit hits the fan, basically, before I ask for any kind of, you know, help and stuff like that. I don’t think it is a good decision, but I just gotta keep my fingers crossed and hopefully nothing happens.*

He went on to say, *“If you see me foaming at the mouth one day, then take me to the doctor, besides then, everything’s ok.”* Cumulative experiences of discrimination, stigmatization, criminalization, and mistrust of the system resulted in major barriers for structurally vulnerable participants to access pain management and end-of-life care in the home.

Additional barriers to providing adequate pain management in the community were also based on a general lack of training, and resulting discomfort, among community health care workers providing end-of-life care for populations actively using, or simply having a history of using, illicit substances (e.g., users’ erratic behaviour/dangerousness; prescribing medications that could result in overdose deaths). A palliative care physician shared that:

*“Most people [community health care workers] feel really uncomfortable, kind of scared around that…they’re worried that people are going to have erratic behaviour, unpredictable and they somehow could endanger the patient or endanger the practitioner or other people around them. So there’s a whole element, all of that fear stuff”.*

Concerns surrounding the lack of training regarding pain management for people actively, or previously, using substances was found to be a significant barrier due to various perceived risks. These perceived risks included those associated with prescribing the levels of medication needed to meet increasing drug tolerances. For example, one housing support worker described, “*especially when you’re using, individuals that use substances, and their understanding of pain, is….like for pain management….they would need much more greater amounts of medication for pain management”.* Even with adequate training, the context of criminalization and stigmatization can prevent health care providers from engaging in meaningful/truthful discussions surrounding substance use with their clients/patients. As a result, prescribing to effectively meet clients’ pain needs is hindered, which increases clients’ risk of pain, suffering, and overdose as people are forced to access a toxic drug supply in order cope. Criminalizing and stigmatizing contexts, along with issues of mistrust within the client/provider relationship, means that many people do not feel safe openly disclosing active or previous use of illicit substances with health care providers. For example, ‘Carl’ shared in an interview that he does not discuss his use of substances with health care providers, even though he describes his reasons for using illicit substances are to cope with his severe pain:

“*I’m just afraid of being judged, you know. Like, people, a lot of people wouldn’t get it, you know, they just wouldn’t get it.”*

The places in which some people live, particularly shelters and supportive housing units, were also found to create barriers for people to adequately manage their pain and symptoms. For example, there were situations observed where the side effects from certain prescription pain management drugs (e.g., severe drowsiness, nausea or vomiting, etc.,) were not conducive to living in more communal/dorm settings. Some participants described skipping their medications because the drowsiness was so heavy, they needed to rest and lie down, but their shelters/housing units did not permit residents to remain in their beds and sleep during the days. One participant also explained how he was unable to keep his pain and seizure medications with him in his room due to the policies of his housing organization, which only allowed medications to be securely held behind the front desk. ‘Carl’ explained:

*I just, I don’t see the mentality on that. You know, they’re non-addictive, they’re not, you know. But hell, you know, they take my meds away from me, what next, you know? … I’m talking important shit here. I have the serious-type seizure… it’s painful enough that where it [pain] comes up my leg, straight up where my pelvis broke. If anybody’s ever had a pain down there like I have, you don’t ever want to have to deal with it. It’s like a sword going straight through me.*

Because ‘Carl’ lived upstairs at his housing organization, once one of these episodes began, he was unable to go down the flights of stairs to access his needed medication. Overall, the complexity surrounding such contexts, including navigating stigma, fear, and lack of training among professionals, minimizing risks of overdose and diversion among active users, and ensuring pain is being effectively managed results in major challenges that are constraining the choices of structurally vulnerable populations to die in their homes.

### Gaps between protocols, policies, and procedures for health teams

While existing protocols, policies, and procedures in housing and community care have been implemented with intentions to meet most people’s care needs, our findings demonstrate that they significantly impede the provision of end-of-life care and the ability for people facing health and social inequities to die-in-place. For example, within a transitional housing facility, a mental health worker commented during a focus group that there is “*a disconnect between upper management and frontline workers*”, who are willing to provide palliative care for tenants and follow their ‘goals of care’. This participant used the example of an existing protocol directing workers to call 911 (emergency responders) if any medical issue arises, even if the person does not want to go to the hospital. A housing worker remarked on how these types of protocols can result in significant harm for some: “*looking at polices, looking at you know ‘this is the way we do things’… these things can really impact people who are structurally vulnerable*”. A shelter worker shared that despite wanting to provide quality palliative care in the community, “*we definitely have places where we hit walls when we want to care and come from that person-centred approach, but organizationally, we can’t”.*

Our findings also highlight how various housing organization policies, such as ‘no guest’ rules and ‘room checks’ and/or cleaning policies resulted in structurally vulnerable participants being at risk of eviction, despite their declining health and having no place else to go. For example, access to informal/family caregiving is typically essential to remain in community as end of life nears. However, ‘no guest’ policies were found to leave participants with no choice but to move, as they were unable to access needed informal care. Furthermore, it was shared by a worker participant that one of their clients who got into a particular housing complex because of their disability, received a notice stating:

*In order to assist us to maintain your building to [organization] standards, we must remind tenants that it is their responsibility to clean up any spills and other accidental occurrences that may affect the cleanliness and maintenance of your building, including all the common hallways, elevators, stairwells, building lobbies, garden areas and parking lots.*

This was highly problematic as their client’s declining health and mobility meant they were unable to physically clean, resulting in them being at risk of eviction. A manager for a supportive housing facility remarked that under current local Residential Tenancy Acts, you “*can’t be evicted for being too sick, but you can be too sick to pass room check, and then they get a 30-day notice* [for eviction]*”.* Many housing units were also found to have particular policies in place that restricted the type of support and care their workers could provide to tenants, reinforcing how their staff were “*support workers, not caregivers”.* Essentially, once care needs became too great, tenants would be identified and evicted. The impacts of eviction were found to be beyond some people’s ability to cope. One outreach worker participant shared how a dying client was going to be evicted but had no where else to go. They described how he was being “*pushed over the edge*” and was strongly considering MAiD (Medical Assistance in Dying) as a solution to his situation. Such findings expose the significant gaps in support that exist and the impacts such housing polices can have for structurally vulnerable populations at the end of life.

Findings indicate that many workers and providers were filling the gaps in supports and services by going beyond protocols, policies, and procedures, in order to provide needed care at home for those positioned as structural vulnerable at the end of life. Such actions were found to place workers in precarious situations since this ‘under the table’ work would often not be formally acknowledged or spoken about, resulting in them not receiving organizational support. Consequences of this care work meant putting their own employment at risk, and if injuries were to occur, being personally liable and financially responsible. For example, some health care providers were observed to be entering ‘restricted no-go’ buildings in order to provide care “*off the grid*”. Health care providers were also observed providing care in environments where policies prohibited them from entering (e.g., where smoking, alcohol, or drug use occurred), and in some cases even giving clients food or money from out-of-pocket. We observed instances of community care workers willing to negotiate safety risks in order to provide needed care, including a community-based nurse who shared that:

*If the client needs you to go and pick up needles at their house, then that’s what you can do. Right? And that’s not a waste of your time or, you know, ‘oh that’s not a nursing role’ which is, in the health authority, you often get that kind of thing – ‘That’s not your job. You shouldn’t be doing that’*.

Although their actions put them at risk for significant professional repercussions, for many it served as their most important coping strategy, aligning a harm-reduction approach to care in their practice, and alleviating the significant emotional and moral distress they experience by bearing witness to suffering on a daily basis. As an outreach worker explained:

*I think sometimes I cope by, like, giving people money for drugs. I know what they are going to spend it on. I knew that’s what they needed. And [local health authority] would probably have fired me if they had known about it and I did it anyways. Because that’s what people needed in order to access the care that they needed in that moment, you know, to not go out for the night and rest their infected feet… to just be inside for the night and have drugs without having to go to work for them.*

Taken together, these findings demonstrate that providers are placing themselves in challenging and sometimes precarious situations as they attempt to provide care to those in need in their homes, despite organizational or institutional policies restricting them from doing so.

## Discussion

Receiving care and dying in a place that feels safe, secure, and allows a person to be surrounded by the people they trust is a key indicator of quality palliative care [1–5]. However, it is critical to recognize how ‘place’ can be experienced differently by diverse population groups, and how social and structural inequity can shape and limit one’s ‘choice’ to die-in-place. For those who experience ongoing harm, institutional settings like hospitals may be particularly triggering and symbolically representative of oppressive systems of control, that produce, reinforce and/or amplify vulnerability, anxiety, fear, and social harms [4, 20, 28]. As such, it is imperative that options allowing structurally vulnerable populations the choice to receive care and die in their own homes, however it is defined by them, exist.

Findings from this analysis indicate that a number of complex barriers are impeding structurally vulnerable populations from dying-in-place. Specifically, these barriers include misaligned perceptions and experiences of risk and safety, issues surrounding the management of pain and suffering in the context of substance use, and the existence of protocols, policies, and procedures implemented by the health and housing systems purported to protect workers, but which create significant impacts for dying structurally vulnerable populations. Together, these findings illuminate how the common rhetoric regarding ‘choice’ in regard to preferred place of death likely exists only for privileged population groups and fails to acknowledge how social and structural forces eliminate choice for others, particularly those who are positioned as structurally vulnerable [2, 4, 10, 44, 45]. Building upon others’ critical research [11, 45, 46], our findings also highlight how dominant discourses of ‘choice’, autonomy, and agency in palliative care may actually serve to reproduce and reify existing inequities. As such, there is a need to reframe current discourse from patient/client ‘choice’, to one that acknowledges the goals of palliative care and the broader socio-political structures that produce inequities [45].

Upon closer examination, it also becomes apparent that current policy directives within Canada, and beyond, that intend to increase supports for home deaths are based upon a number of environmental assumptions that reflect normative definitions of ‘home’. Within this discourse, ‘home’ is defined as a physical, bounded dwelling, that meets the conditions identified by community care organizations as safe and secure for workers to enter, and where family caregivers are able to stay to provide needed informal end-of-life care [1, 21, 47–49]. The notion of ‘home’ continues to be used uncritically, and fails to be acknowledged for its complexity, fluidity, and the various ways ‘home’ may be defined for different population groups [4, 7]. The home is a complex environment where political, physical, emotional, social, and cultural elements converge, shaping access to care [50–52]. Essentially, our findings demonstrate that the desire to die-in-place, when the ‘home’ does not align with traditional societal views of what a home is, has resulted in significant tensions and barriers in access to care. The outcome is that despite structurally vulnerable populations not ‘fitting’ into public/formal healthcare environments, without access to home care, they simply have no place else to go to.

### Implications for policy and practice

Taken together, our findings emphasize how access to palliative home care, and the ability to be cared for and die-in-place, are shaped by forces external to individual health care choices and interactions. For example, the ways in which notions of home, risk, and safety are defined and services organized can determine the amount of work required from people to ‘fit’ into those services [45, 53]. Practical efforts and strategies to improve equitable access to palliative care should, therefore, acknowledge the structural forces perpetuating inequities and the power that differing populations groups have to act within them. By shifting the lens of focus from individual professional and client responsibilities, expectations, and choices, to the structures, systems, and institutions, efforts will have greater potential to bring about change, achieve more equity-oriented policies and practices, and ultimately enhance the opportunity for all to ‘die well’ [11, 45, 53].

Simultaneously, on the ground, our findings highlight the value of enhancing supports, education, and training for community care workers regarding the provision of care for structurally vulnerable populations. Greater acknowledgement regarding the subjective notions of risk and safety, rather than the implementation of ‘blanket’ policies, would also enhance access to, and quality of, care. For example, integrating a system that would allow individual workers the autonomy to define their own risk thresholds and the population groups they feel comfortable working with, would enhance safety for all, not only workers, but clients as well. Integrating palliative approaches to care into the everyday work of non-health care providers is also needed as it would enhance collaboration between all providers, fostering trust and the provision of meaningful care in the places that populations experiencing structural vulnerability want to live and die in [19]. Finally, by acknowledging, valuing, and building upon existing relations of care, including formal and informal care networks, the current challenges of delivering palliative care in settings with inadequate support can be reduced, while increasing ‘choice’ for all people at their end of life [2, 21].

### Strengths and limitations

The studies included in this analysis were not focused on exploring barriers or challenges to dying-in-place for people positioned as structurally vulnerable. Rather, barriers and challenges for structurally vulnerable populations at the end of life to remain at home emerged throughout our research and analysis as a significant finding. Therefore, while this analysis begins to shed light on these complex issues, more research is needed in order to garner a deeper understanding on what dying-in-place means in the context of structural vulnerability. Furthermore, the research data were collected in only one urban centre in Canada. As such, similar data is needed to be collected in other communities located in various geographic contexts in order to gain a greater understanding of the diversity of experiences that exist. Finally, although the data for this project were collected back in 2014–2019, our team has since continued to engage with inner-city workers and palliative care practitioners and decision-makers to integrate palliative approaches to care for structurally vulnerable populations in this community. This research has been instrumental in the development, implementation, and evaluation of the Palliative Outreach Resource Team (PORT), a mobile, palliative care team serving people facing life-limiting conditions alongside poverty, homelessness, racism, and discrimination.

## Conclusion

A central measure of quality in palliative care is to ensure the wishes and desires of people approaching the end of life are acknowledged and realized, particularly in regard to where they would like to be to receive care, and ultimately, die. Current palliative care policy directives within Canada, and beyond, however, fail to recognize how ‘choice’ is only available to privileged populations groups [44]. As our findings begin to demonstrate, without acknowledging the various ways that social and structural factors restrict actual opportunities, such discourses of choice only serve to reinforce inequities for populations positioned as structurally vulnerable. Considering the multitude of ways that structurally vulnerable populations are excluded, stigmatized, and harmed within traditional health care systems and settings (i.e., hospitals), it becomes imperative that the options to die-in-place (e.g., in their homes/communities where they feel safe), becomes critically important. This, however, not only requires a re-evaluation of the definitions of ‘home’ within palliative care, but also the need to acknowledge the contexts in which many people live that do not ‘fit’ within the more traditional, normative, structures upon which our health care systems are based [26, 29–31]. Finally, although our research sheds light on some of the barriers that exist, it is important to also acknowledge the strides that have been made regarding successful interventions for a palliative approach to care for structurally vulnerable populations. Some examples of these include: [1] equity-oriented mobile palliative teams providing care where people are at and a bridging with healthcare services (e.g. Palliative Outreach Resource Team [PORT] [54]; Community Allied Mobile Palliative Partnership [CAMPP] [55]; Palliative Care Outreach and Advocacy Team [PCOAT] [56]; and Palliative Education And Care for the Homeless [PEACH] [57]); equity-oriented residential hospices (e.g., Ottawa Mission [58]); equity-oriented policies and practices within mainstream hospice and palliative care units and services; and integration of a palliative approach to care in the places where people are living and dying in contexts of inequity (e.g., shelters, supportive housing, etc.). Despite these advances, however, inequities still persist. As such, a dire need exists to continue working towards enhancing inclusivity within palliative care policy, programs, and practice to ensure all people have the ‘choice’ and opportunity to die with dignity in the places that they prefer.

### Electronic supplementary material

Below is the link to the electronic supplementary material.

Supplementary Material 1: Observation and Focus Group Guide for Equitable Access to Care (EAC) study

Supplementary Material 2: Observation and Focus Group Guide for Integrating a Palliative Approach to Care in the inner-city (iPAC-IC)

## Data Availability

The datasets generated and/or analysed during the current study are not publicly available to protect study participant privacy, but are available from the corresponding author on reasonable request.

## References

[1] Canadian Institute for Health Information. Access to palliative care in Canada 2018 [Available from: https://www.cihi.ca/sites/default/files/document/access-palliative-care-2018-en-web.pdf

[2] Grindrod A (2020). Choice depends on options: a public health framework incorporating the social determinants of dying to create options at end of life. Progress in Palliative Care.

[3] van Doorne I, van Rijn M, Dofferhoff SM, Willems DL, Buurman BM (2021). Patients’ preferred place of death: patients are willing to consider their preferences, but someone has to ask them. Age Ageing.

[4] Collier A, Broom A (2021). Unsettling place(s) at the end of life. Soc Sci Med.

[5] Zaman M, Espinal-Arango S, Mohapatra A, Jadad AR (2021). What would it take to die well? A systematic review of systematic reviews on the conditions for a good death. The Lancet.

[6] World Health Organization. Palliative Care 2020 [Available from: https://www.who.int/news-room/fact-sheets/detail/palliative-care

[7] Driessen A, Borgstrom E, Cohn S (2021). Placing death and dying: making place at the end of life. Soc Sci Med.

[8] Turner V, Flemming K (2019). Socioeconomic factors affecting access to preferred place of death: a qualitative evidence synthesis. Palliat Med.

[9] Canadian Home Care Association (2006). Pan-canadian gold standard for palliative home care: toward equitable access to high quality hospice palliative and end-of-life care at home.

[10] Borgstrom E, Walter T (2015). Choice and compassion at the end of life: a critical analysis of recent English policy discourse. Soc Sci Med.

[11] French M, Hansford L, Moeke-Maxwell T (2023). Reflecting on choices and responsibility in palliative care in the context of social disadvantage. Palliat Care Social Pract.

[12] Bondi L (2008). On the relational dynamics of caring: a psychotherapeutic approach to emotional and power dimensions of women’s care work. Gend Place Cult.

[13] Reimer-Kirkham S, Stajduhar K, Pauly B, Giesbrecht M, Mollison A, McNeil R (2016). Death is a social justice issue: perspectives on equity-informed palliative care. Adv Nurs Sci.

[14] Stajduhar KI, Mollison A, Giesbrecht M, McNeil R, Pauly B, Reimer-Kirkham S (2019). Just too busy living in the moment and surviving: barriers to accessing health care for structurally vulnerable populations at the end-of-life. BMC Palliat care.

[15] Stajduhar K, Mollison A (2018). Too little, too late: how we fail vulnerable canadians as the die and what to do about it.

[16] Bourgois P, Holmes SM, Sue K, Quesada J (2017). Structural vulnerability: operationalizing the Concept to address Health disparities in Clinical Care. Acad Med.

[17] McNeil R, Kerr T, Anderson S, Maher L, Keewatin C, Milloy MJ (2015). Negotiating structural vulnerability following regulatory changes to a provincial methadone program in Vancouver, Canada: a qualitative study. Soc Sci Med.

[18] Quesada J, Hart LK, Bourgois P (2011). Structural vulnerability and health: latino migrant laborers in the United States. Med Anthropol.

[19] Stajduhar KI, Giesbrecht M, Mollison A, d’Archangelo M (2020). Everybody in this community is at risk of dying: an ethnographic exploration on the potential of integrating a palliative approach to care among workers in inner-city settings. Palliat Support Care.

[20] Giesbrecht M, Stajduhar K, Mollison A, Pauly B, Reimer-Kirkham S, McNeil R (2018). Hospitals, clinics, and palliative care units: place-based experiences of formal healthcare settings by people experiencing structural vulnerability at the end-of-life. Health Place.

[21] Stajduhar KI, Giesbrecht M, Mollison A, Dosani N, McNeil R (2020). Caregiving at the margins: an ethnographic exploration of family caregivers experiences providing care for structurally vulnerable populations at the end-of-life. Palliat Med.

[22] Browne AJ, Varcoe CM, Wong ST, Smye VL, Lovoie J, Littlejohn D et al. Closing the health equity gap: evidence-based strategies for primary health care organizations. Int J Equity Health. 2012;1(59). 10.1186/1475-9276-11-59 PMC3570279 23061433

[23] Klop HT, Evenblij K, Gootjes JRG, de Veer AJE, Onwuteaka-Philipsen BD (2018). Care avoidance among homeless people and access to care: an interview study among spiritual caregivers, street pastors, homeless outreach workers and formerly homeless people. BMC Pub Health.

[24] Sumalinog R, Harrington K, Dosani N, Hwang SW. Advance care planning, palliative care, and end-of-life care interventions for homeless people: a systematic review. Palliat Med. 2016;Online first: June 3, 2016. 10.1177/0269216316649334 27260169

[25] Moller DW (2012). Dancing with broken bones: poverty, race, and spirit-filled dying in the inner city.

[26] Ko E, Kwak J, Nelson-Becker H (2015). What constitutes a good and bad death? Perspectives of homeless older adults. Death Stud.

[27] Collier R (2011). Bringing palliative care to the homeless. CMAJ.

[28] Podymow T, Turnbull J, Coyle D (2006). Shelter-based palliative care for the homeless terminally ill. Palliat Med.

[29] Song J, Bartels D, Ratner E, Alderton L, Hudson B, Ahluwalia J (2007). Dying on the streets: homeless persons’ concerns and desires about end of life care. J Gen Inter Med.

[30] Krakowsky Y, Gofine M, Brown P, Danziger J, Knowles J (2013). Increasing access—A qualitative study of homelessness and palliative care in a major urban center. Am J Hospice Palliat Care.

[31] Håkanson C, Sandberg J, Ekstedt M, Sarenmalm EK, Christiansen M, Öhlén J (2016). Providing Palliative Care in a Swedish support home for people who are homeless. Qual Health Res.

[32] Stajduhar KI, Nickel DD, Martin WL, Funk L (2008). Situated/being situated: client and co-worker roles of family caregivers in hospice palliative care. Soc Sci Med.

[33] Savage J (2000). Ethnography and health care. BMJ.

[34] Schensul S, Schensul L, LeCompte JJ (1999). D. enhanced ethnographic methods.

[35] Fetterman D, Ethnography M (2003). Encyclopedia of Social Science Research Methods.

[36] Thomas J (2003). Critical ethnography. Encyclopedia of Social Science Research Methods.

[37] Tremblay M-C, Martin DH, McComber AM, McGregor A, Macaulay AC (2018). Understanding community-based participatory research through a social movement framework: a case study of the Kahnawake Schools Diabetes Prevention Project. BMC Public Health.

[38] Creswell JW (1998). Qualitative Inquiry and Research Design: choosing among five traditions.

[39] Giesbrecht M, Mollison A, Whitlock K, Stadjuhar K. Once you open that door, it’s a floodgate: exploring work-related grief among community service workers providing care for structurally vulnerable populations at the end of life through participatory action research. Palliat Med. 2022;37(4). 10.1177/02692163221139727 PMC10074740 36461158

[40] Equity in Palliative Approaches to Care Collaborative. Equity-informed advance care planning 2020 [Available from: https://www.equityinpalliativecare.com/acpresources

[41] Peterson BL. Thematic Analysis/Interpretive Thematic Analysis. In: Matthes J, Davis CS, Potter RF, editors. The International Encyclopedia of Communication Research Methods2017. p. 1–9.

[42] Stern PN, Given LM (2008). Constant comparison. Sage encyclopedia of qualitative research methods.

[43] Given LM, Saumure K, Triangulation, Given LM (2008). Sage encyclopedia of qualitative research methods.

[44] Stajduhar KI (2020). Provocations on privilege in palliative care: are we meeting our core mandate?. Progress in Palliative Care.

[45] French M, Keegan T, Preston N (2023). Facilitating equitable access to hospice care in socially deprived areas: a mixed methods multiple case study. Palliat Med.

[46] Sutherland N, Ward-Griffin C, McWilliam C, Stajduhar K (2018). Discourses reproducing gender inequities in Hospice Palliative Home Care. Can J Nurs Res.

[47] Giesbrecht MD, Crooks VA, Williams A, Hankivsky O. Critically examining diversity in end-of-life family caregiving: implications for equitable caregiving support and Canada’s Compassionate Care Benefit. Int J Equity Health. 2012;11(65). 10.1186/1475-9276-11-65 PMC3502300 23116474

[48] Carstairs S, MacDonald ML. The PRISMA Symposium 2: Lessons From Beyond Europe. Reflections on the Evolution of Palliative Care Research and Policy in Canada. Journal of Pain and Symptom Management. 2011;42(4):501-4. 10.1016/j.jpainsymman.2011.06.009 21872425

[49] Canadian Hospice Palliative Care Association (2012). Fact sheet: hospice and palliative care in Canada.

[50] Milligan C (2012). There’s no place like home: place and care in an ageing society.

[51] England K (2010). Home, work and the shifting geographies of care. Ethics Place & Environmnet: A Journal of Philosophy & Geography.

[52] Exley C, Allen D (2007). A critical examination of home care: end of life care as an illustrative case. Soc Sci Med.

[53] Dixon-Woods M, Cavers D, Agarwal S, Annandale E, Arthur A, Harvey J (2006). Conducting a critical interpretive synthesis of the literature on access to healthcare by vulnerable groups. BMC Med Res Methodol.

[54] Equity in Palliative Approaches to Care. Palliative Outreach Resource Team (PORT) 2023 [Available from: https://www.equityinpalliativecare.com/port

[55] Community Allied Mobile Palliative Partnership (CAMPP). Community Allied Mobile Palliative Partnership 2023 [Available from: http://www.campp.ca/

[56] Alberta Medical Association. Alberta Doctors’ Digest: Dr. Carla Bablitz 2023 [Available from: https://add.albertadoctors.org/profiles/dr-cara-bablitz/

[57] Inner City Health Associates. PEACH - Palliative Education and Care for the Homeless 2023 [Available from: https://www.icha-toronto.ca/programs/peach-palliative-education-and-care-for-the-homeless

[58] The Ottawa Mission. The Ottawa Mission 2023 [Available from: https://ottawamission.com/

[59] O’Brien B, Harris I, Beckman T, Reed D, Cook D (2014). Standards for reporting qualitative research: a synthesis of recommendations. Acad Med.

