# Supplementary material for: “You can’t die here”: an exploration of the barriers to dying-in-place for structurally vulnerable populations in an urban centre in British Columbia, Canada
Source: BMC Palliat Care. 2024 Jan 10;23:12. doi: 10.1186/s12904-024-01340-7 (PMC10782732; doi:10.1186/s12904-024-01340-7)
Supplement: Supplementary file 1 — Supplementary Material 1: Observation and Focus Group Guide for Equitable Access to Care (EAC) study [file 12904_2024_1340_MOESM1_ESM.pdf]

Supplementary Material 1: Observation and Focus Group Guide for Equitable Access to Care (EAC) study

Ethnographic Observation Guide:

These are the types of things you will want to make note of and describe in as much detail as possible in your fieldnotes:

- Describe where the observation takes place (e.g., in public spaces, in private rooms, health clinic, hospital, shelter, home, etc.). If the observation occurs in the structurally vulnerable person's home, carefully describe their living arrangements (what is the layout of the home, is there a bathroom, what does it include, what floor do they live on, is there an elevator in the building.) After an observation try to draw a birds-eye view map of the observation location.
- Describe the setting at the time of observation (e.g. in clinic settings: who is present, what time of day is it, is it busy/quiet; in other settings: who is present, what are the surroundings, layout of the space, etc.)
- Describe who is involved during encounters between structurally vulnerable people and care/service providers (e.g., patient, other family members/friends, other providers, etc.)
- What are the participants' relationships with each other? Does the structurally vulnerable person have a support person? Do they live with them? Are there others in their social circle who may play a role in their care? (e.g. help them get to and from services, bring them meds, food, etc).
- Make note of who interacts with each other and what the interactions involve.
    - Where do they take place and what are the topics of discussion?
    - How do the service providers assess and address the structurally vulnerable person's needs?
    - What kinds of barriers do the structurally vulnerable people encounter (e.g. access is barred because of substance use, difficulty getting to and from services, etc) in accessing end of life care? What are the facilitators?
    - What role does the support person play in the structurally vulnerable person's access to end of life care?
- Detail the aspects of the interactions.
    - How long is the interaction?
    - Were there comprehension barriers?
    - Who initiates/ends conversations? How are questions asked and answered? For instance, who initiates conversations around care?
    - How are care needs addressed? Describe how clinical decisions are raised and discussed, and how decision-making occurs. For instance, to what extent are certain decisions "negotiated" and how?

- Describe interactions between the structurally vulnerable person and others in the setting (what is the tone of interactions, verbal or behavioral cues that influence the interaction)
- Describe any non-verbal communication patterns, any physical contact between the structurally vulnerable person and care providers or support persons (e.g., gestures of support or comfort such as touching one's shoulder)
- Describe the physical position of the structurally vulnerable person and the position of the care provider or others during interactions (e.g., sitting down facing each other, standing in the hallway)
- Whenever possible, capture verbatim accounts from participants. Describe the informal conversations that occur between yourself and the structurally vulnerable person and/or care providers and/or family
- Detail the structurally vulnerable participant's health issues that come up during the observation. List conditions; describe their physical and mental state.

The following are some types of secondary information that you might be able to gather through your observations and that should be noted:

- If the structurally vulnerable participant does not have housing, where do they spend the majority of their time, where do they sleep?
- During observations, make note of housing arrangements, and socio-demographic information that may not have been captured by the demographic form, but that appears to influence care and access to care.
- How does the structurally vulnerable participant (and their caregiver) get around (walk? bus? bike?).
- Make note of anything else that strikes you as important to the study topic or as influencing what you observed.

Semi-Structured Interview Guides:

People experiencing structural vulnerability:

1. We're interested in understanding your experiences accessing health care, so to begin can you please tell me about your care needs (in the past month/year)?
   Prompts:
   * What types of health conditions do you have?
   * What kind of support do you need (e.g. medication, medical attention, housing, nutrition, etc.)
2. When you need medical assistance how and where do you get it?
   Prompts:
   * For minor or non-emergency type issues
   * For immediate or severe health issues
3. Have you had any negative interactions with health care providers? If so can you explain?

4. Have you had any positive interactions with health care providers? If so can you explain?
5. What barriers do you see in terms of getting access to health care that you need?
   Prompts:
   * Options for accessing care
   * Transportation or getting to and from care
   * Rules associated with use of services (e.g. substance use policies)
6. Is there anything that makes access to health care easier?

Caregivers/supporters:

1. We're interested in understanding your experiences caring for and assisting [client] in accessing health care, so to begin how did you start caring for [client]?
2. What types of things do you help them with?
3. What barriers do you see in terms of getting access to health care that [client] needs?
   Prompts:
   * Options for accessing care
   * Transportation or getting to and from care
   * Rules associated with use of services (e.g. substance use policies)
4. Is there anything that makes access to health care easier?
5. What types of barriers do you face in caring for [client]
6. What types of things make it easier for you to get health care for [client]

Service providers:

1. What are some of the main health issues that homeless and unstably housed members of the community with life limiting conditions face?
   Prompts:
   * In terms of managing their conditions?
2. What barriers are there for this population in accessing the health care that they need?
3. What makes their access to health care easier?
4. What organizational structures are in place to support this population's access to care?
5. What are the barriers or gaps in the organizational structure that inhibit this population's access to care?
6. What interventions, health services and policies will best promote access to equitable care for this population?
