# Supplementary material for: “You can’t die here”: an exploration of the barriers to dying-in-place for structurally vulnerable populations in an urban centre in British Columbia, Canada
Source: BMC Palliat Care. 2024 Jan 10;23:12. doi: 10.1186/s12904-024-01340-7 (PMC10782732; doi:10.1186/s12904-024-01340-7)
Supplement: Supplementary file 2 — Supplementary Material 2: Observation and Focus Group Guide for Integrating a Palliative Approach to Care in the inner-city (iPAC-IC) [file 12904_2024_1340_MOESM2_ESM.pdf]

Supplementary Material 2: Observation and Focus Group Guide for Integrating a Palliative Approach to Care in the inner-city (iPAC-IC)

Action Team Baseline Focus Group Guide:

1. Give an example of a person who was housed in your setting who was very sick and frail and who you thought might be dying. What made you think that they might be dying?
2. What affected your ability to provide the kind of care and support you wished to?
3. How do you currently care for people who you think might be dying? How does care for this person typically unfold?
4. What do you focus on when providing care?
5. What education related to palliative care have you had?
6. What areas of education do you think would be helpful to enhance your capacity to care?
7. How satisfied are you with the care you are able to provide? What influences this?
8. Are there any things that get in the way of you being able to provide the support and care you think is required? If so, what gets in the way?
9. Are there any things that help you to be able to provide the support and care you think is required? If so, what are those things that help?
10. Is there anything else you would like to share that will help us better understand how to support you in your work when caring for people who could benefit from a palliative approach?

Action Team Structured Report Guide:

1. What ideas did you take away from the session?
2. How will these ideas impact your work?
3. What strategies might you try out between now and the next strategy session?

Action Team Observation Guide:
These are the types of things you will want to make note of and describe in as much detail as possible in your fieldnotes:

- Describe the setting at the time of observation (e.g. who is present, what time of day is it, is it busy/quiet; in other settings: who is present, what are the surroundings, layout of the space, etc.)
- Describe who is involved in the Action Team and what kinds of activities make up their everyday work. What are their work contexts? What are the participants' relationships with each other?
- Make note of who interacts with each other and what the interactions involve.
  - What are the topics of discussion?
  - How do the service providers assess and address the structurally vulnerable person's needs?
  - What kinds of barriers do the service providers come up against in caring for those at end-of-life? What are the facilitators?
  - What role do the service providers play in the structurally vulnerable person's access to end of life care?
- Make notes on discussions of a palliative approach to care.
  - What are some barriers to implementing a palliative approach in the workplaces of these inner city workers?
  - What kinds of commonalities exist in the educational needs and practice support tools required for adaptation for a palliative approach?
  - What strategies are emerging as possible approaches (e.g., education, consciousness raising, adaptation of available practice support tools, adjustment of work flow)?
- How is knowledge and experience changing through the Action Cycles?
- Whenever possible, capture verbatim accounts from participants. Describe the informal conversations that occur between the service providers

Action Team Summative Focus Group Guide:

1. Has your involvement with/in the Action Cycles impacted your work in any way? Can you give us an example?

2. Do you feel like you have a better understanding of palliative approaches to care? Can you give us an example?

3. Are you better able to identify people who could benefit from palliative approaches to care?

4. What stands out to you as the most important learning? Can you tell us of a time where you put this learning into practice?

5. What areas of education do you think would be still be helpful to enhance your capacity to care?

6. Are there things that still get in the way of you being able to provide the support and care you think is required? If so, what gets in the way?

7. Are there things that you plan to do going forward to continue to build capacity in your organization for integrating a palliative approach?

8. Do you have anything else to share from your experience in this project?
